# Optimization of Crystal Structures in Polylithionite Concentrate: A Molecular Dynamics Approach to Lithium Extraction Efficiency

**DOI:** 10.3390/nano14211713

**Published:** 2024-10-27

**Authors:** María Guadalupe Quezada-Aldaco, Efren Delgado, David Enrique Zazueta-Álvarez, Víctor Jesús Martínez-Gómez, Hiram Medrano-Roldán, Perla Guadalupe Vázquez-Ortega, Felipe Samuel Hernández-Rodarte, Damián Reyes-Jáquez

**Affiliations:** 1 Department of Chemical and Biochemical Engineering, National Technological Institute of Mexico (TecNM)–Durango Institute of Technology (ITD), Blvd. Felipe Pescador 1830, Nueva Vizcaya, Durango 34080, Mexico; 22040317@itdurango.edu.mx (M.G.Q.-A.); v.martinez@itdurango.edu.mx (V.J.M.-G.); hmedrano@itdurango.edu.mx (H.M.-R.); pvazquez@itdurango.edu.mx (P.G.V.-O.); shernandez@itdurango.edu.mx (F.S.H.-R.); 2 Food Science and Technology, Department of Family and Consumer Sciences, New Mexico State University, P.O. Box 30001, Las Cruces, NM 88003-8001, USA; edelgad@nmsu.edu; 3 Ingeniería en Tecnología Ambiental, Universidad Politécnica de Durango, Carretera Durango-México Km 9.5, Durango 34300, Mexico; david.zazueta@unipolidgo.edu.mx

**Keywords:** mining, geometric optimization, structural validation, miscibility

## Abstract

Molecular dynamics (MD) techniques offer significant potential for optimizing mineral extraction processes by simulating economically or physically restrictive conditions at the laboratory level. Lithium, a crucial metal in the electromobility era, exemplifies the need for ongoing re-evaluation of extraction techniques. This research aims to simulate the crystal structures of mineral species present in a polylithionite mineral concentrate [KLi_2_Al(Si_4_O_10_)(F,OH)_2_] using crystallographic data obtained from X-ray diffraction analysis. This study focuses on optimizing these structures, validating them through density comparisons, and determining the interaction parameter between the identified phases and lithium oxide (Li_2_O). The X-ray diffraction analysis revealed five predominant mineral phases: quartz (SiO_2_), calcite [Ca(CO_3_)], pyrite (FeS_2_), cassiterite (SiO_2_), and a compound Pb_6_O_2_(BO_3_)_2_SO_4_. Structural data, including lattice parameters, space groups, and atomic coordinates, were used to construct the crystal structures with Materials Studio 8.0, employing the Crystal Builder module. Optimization was performed using the Forcite module with the Smart optimization algorithm and the Universal force field. The interaction parameter (χ) indicated an affinity between lithium oxide and pyrite, as well as between calcite and quartz.

## 1. Introduction

Molecular dynamics (MD) simulation techniques have become essential tools in research across various fields, including biomolecules, polymers, minerals, and other compounds. These techniques are used to test theoretical models and predict experimental behaviors such as interactions and structural dynamics. By describing variations in positions, velocities, and orientations of molecules over time, MD simulations facilitate a deeper understanding of these systems [1,2].

When implementing MD simulations, researchers often rely on complementary analytical techniques to enhance accuracy and efficiency. Techniques such as X-ray crystallography [3], electron microscopy [4], nuclear magnetic resonance (NMR) [5], and electron paramagnetic resonance (EPR) are commonly used to support and refine simulation results [2].

In the mining industry, MD simulations have diverse applications. They can aid in the development of new compounds and structures and simulate interactions between minerals to assess the stability of molecular complexes under varying pressure and temperature conditions [6]. MD simulations can also evaluate the behavior of compounds or crystalline structures under different pressures [7] and determine formation energies at various temperatures [8]. By simulating laboratory conditions that might be challenging to replicate experimentally due to economic or physical constraints, MD techniques offer valuable insights [9].

In recent decades, focus has shifted towards exploring compounds and elements that can aid in the decarbonization of energy sources and address current energy demands. Energy storage and production technologies, such as rechargeable batteries [10], electric vehicles [11], and storage cells [12], are key areas of exploration. Elements like lithium, cobalt, zinc, and magnesium are being investigated as crucial components of energy transition [13].

Mining companies are becoming increasingly interested in developing sustainable technologies to reduce the environmental impact of mineral extraction. Biohydrometallurgy, a branch of biotechnology, explores the economic potential of microbial interactions with mineral compounds while addressing environmental concerns [14]. Molecular dynamics simulations might enhance the efficiency of mineral extraction processes by generating predictive models for ore deposits and optimizing biotechnological treatments [13].

This research focused on simulating the crystalline structures of mineral species present in lithium concentrate from Bacanora Minerals, located in Bacadehuachi, Sonora, Mexico. This study aimed to optimize these structures using X-ray diffraction (XRD) analysis, allowing for a comparison between simulated structural approximations and those observed in the mineral concentrate. Additionally, this research assessed the miscibility parameters between the phases identified through XRD analysis and lithium oxide (Li_2_O) to evaluate their affinity. This research could serve as a foundation for in silico evaluations that might otherwise be economically prohibitive, offering significant benefits to the mining industry and related lithium sectors.

## 2. Materials and Methods

### 2.1. Materials

The lithium-bearing mineral concentrate was a clay mineral identified as polylithionite [KLi_2_Al(Si_4_O_10_)(F,OH)]_2_ [15]. This material was supplied by Bacanora Minerals, a company located in Sonora, northeastern Mexico, approximately 11 km south of Bacadehuachi, 180 km northeast of Hermosillo, and about 170 km south of the US–Mexico border.

### 2.2. Methods

#### 2.2.1. X-Ray Diffraction Analysis

The morphology of the mineral concentrate was assessed using X-ray diffraction (XRD) analysis. The sample was crushed and sieved to obtain particles smaller than 200 mesh. It was then homogenized to ensure uniform exposure to the X-ray beam. A Rigaku Miniflex 600 X-ray (Rigaku Corp., Tokyo, Japan) diffractometer was used, operating at room temperature with a monochromatic Cu Kα radiation source (λ = 0.154 nm). The analysis was conducted in stepped scan mode with a 2θ angular range of 5–90° and a step size of 0.02°.

#### 2.2.2. Crystal Builder

To construct the crystal structures present in the mineral concentrate, we used the Crystal Builder module of Materials Studio 8.0. This involved inputting structural parameters derived from the XRD analysis, such as lattice parameters, space groups, angles, and the coordinates of the constituent atoms for each crystal phase.

#### 2.2.3. Geometry Optimization

Geometric optimization of each structure was performed through an energetic evaluation and conformational adjustment. Atomic coordinates and cell parameters were refined until the structure reached its minimum energy state. This was accomplished using the Forcite module [16] with the Smart optimization algorithm [17], which combines various geometry optimization methods to better approximate minimum energy potential. The Universal force field, which incorporates rules based on hybridization and connectivity, was used to calculate forces on atoms from their potential energy. This force field is suitable for complex metallic compounds and predicts geometries and conformational energy differences while considering the crystalline nature of the sample [18].

#### 2.2.4. Structural Validation

Structural validation of the crystals involved comparing the densities of simulated structures with those obtained experimentally. Structural densities are closely related to intermolecular optimizations [19,20]; a higher percentage of agreement indicates greater structural accuracy [21,22]. Experimental densities were obtained from X-ray diffraction analysis, while simulated densities were calculated using Materials Studio 8.0. The percentage of agreement between experimental and simulated densities was then computed.

#### 2.2.5. Miscibility

An atomic absorption analysis previously conducted on the mineral concentrate samples revealed a lithium concentration of 0.0052%. Given this concentration, lithium oxide was not detected among most species in the X-ray diffraction analysis, as the equipment requires a minimum concentration of 0.5% lithium. Therefore, we employed dynamic simulation techniques to identify the likely predominant entity or species responsible for lithium release. This was carried out using the Blends module of Materials Studio 8.0, which considers the miscibility parameter. The Blends module uses the Flory–Huggins model, a well-known theory of the thermodynamics of mixing and phase separation in binary systems [23]. The general expression for the free energy of mixing is given by Equation (1) [24].

(1) $$\frac{∆G}{RT}=\frac{\emptyset_{b}}{n_{b}}ln\emptyset_{b}+\frac{\emptyset_{s}}{n_{s}}ln\emptyset_{s}+\chi \emptyset_{b}\emptyset_{s}$$

where 

∆G= Free energy of mixing per mole $\left(\frac{∆G}{mol}\right)$.

$\emptyset_{i}=$ Volume fraction of component i.

$n_{i}=$ Degree of polymerization of component *i*.

χ= Interaction parameter. 

T= Absolute temperature (K).

R= Universal gas constant $\left(\frac{J}{mol*K}\right)$.

In the model, the first term represents the combinatorial entropy of the system. This term is typically negative, which favors the formation of a mixed state in pure compounds. A more negative combinatorial entropy value indicates a stronger attraction between the interacting elements as they form a complex [25].

Conversely, the last term represents the free energy of mixing resulting from the interaction. If the interaction parameter, χ, is positive, it disfavors the mixed state due to the degrees of freedom and the restricted miscibility of the elements in the mixing complex, which tend to segregate [25]. The interaction parameter (χ) is defined by Equation (2).

(2) $$\chi =\frac{E_{mix}}{RT}$$

where $E_{mix}=$ mixing energy, i.e., the difference in free energy for the interaction between the mixed state and the pure state. 

In the traditional Flory–Huggins model, each mixing component occupies a lattice site. In such a lattice with a coordination number *Z*, binding energy represents the interaction energy between two components. This interaction energy allows for the calculation of mixing energy, the interaction parameter *χ*, and the construction of phase diagrams [25]. Mixing energy is described by Equation (3).

(3) $$E_{mix}=\frac{1}{2}Z(E_{bs}+E_{sb}-E_{bb}-E_{ss})$$

where $E_{ij}$ ${(E}_{bs},E_{sb},E_{bb},E_{ss})$ represent the binding energy between a unit of the component and a unit of the component i and a unit of the component j.

On the other hand, the Blends module generates multiple molecular orientations by calculating the interaction energy for each configuration. These orientations are classified based on their assigned roles, which are termed the “base role” and “screen role.” There are four possible combinations of these roles, each with an associated binding energy value: base–base (*E_bb_*), screen–screen (*E_ss_*), screen–base (*E_sb_*), and base–screen (*E_bs_*) [26]. The mixing energy at a given temperature is represented by Equation (4).

(4) $$E_{mix}=\frac{1}{2}(Z_{bs}{\langle E_{bs}\rangle }_{T}+Z_{sb}{\langle E_{sb}\rangle }_{T}-Z_{ss}{\langle E_{ss}\rangle }_{T})$$

A value of χ ≤ 0 indicates that, at a given temperature, the molecules interact favorably, allowing the mixture to potentially exhibit a single phase. Conversely, if χ ≥ 0, it suggests that the molecules have a preference for being surrounded by similar components rather than mixing. Finally, if χ ≫ 0, this indicates that the contribution to free energy surpasses the combinatorial entropy of the mixture, leading to phase separation into two distinct phases.

## 3. Results

### 3.1. X-Ray Diffraction Analysis

**Figure 1:**
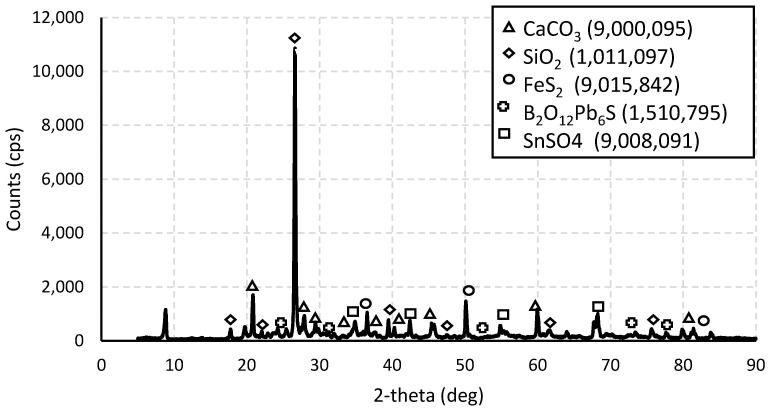
Diffraction pattern of the mineral sample with lithium and its majority species.

The results of the XRD analysis are shown in Figure 1. The data reveal the presence of five predominant species. According to the quantitative Rietveld analysis (RIR) performed by the equipment, the most abundant compound is quartz (SiO_2_), constituting 92% of the sample. This is followed by calcite [Ca(CO_3_)], at 5.4%. Additionally, the analysis identified pyrite (FeS_2_), tin oxide (SnO_2_), and a compound with the formula Pb_6_O_2_(BO_3_)_2_SO_4_.

### 3.2. Crystal Builder 

The XRD analysis provided crystallographic data, including space groups, lattice parameters (Table 1), and atomic positions within the crystal lattice (Table 2). This information was utilized to construct crystals using the Crystal Builder module in Materials Studio 8.0 software.

**Figure 2:**
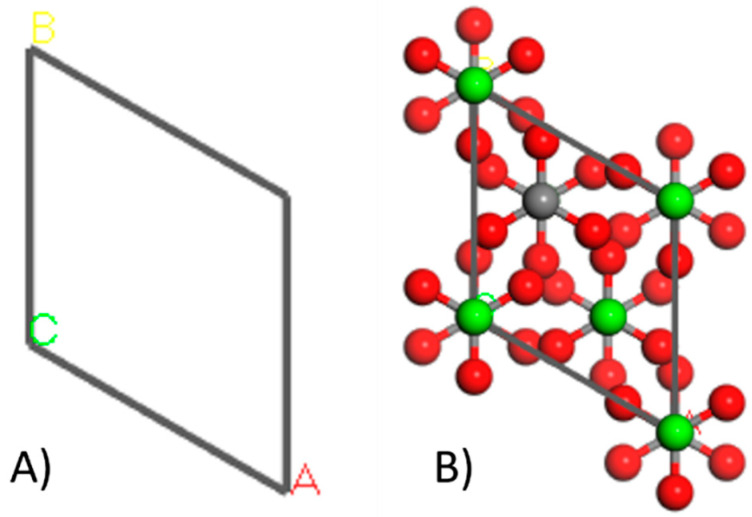
Representation of the unit cell corresponding to the construction of a calcite crystal (**A**) and representation of the calcite crystal after adding the atoms in the indicated positions (**B**).

The first step in constructing the crystals was defining the unit cell, starting with the information provided by the XRD analysis on lattice parameters, including size and corresponding angles [28] (Table 1, Figure 2). Next, the coordinates of the atoms were inputted to specify their positions within the cell (Table 2). For the creation of the lithium oxide (Li_2_O) crystal, structural data such as lattice parameters, space groups, and atomic coordinates were obtained from The “Materials Project” database [27].

### 3.3. Geometry Optimization 

**Figure 3:**
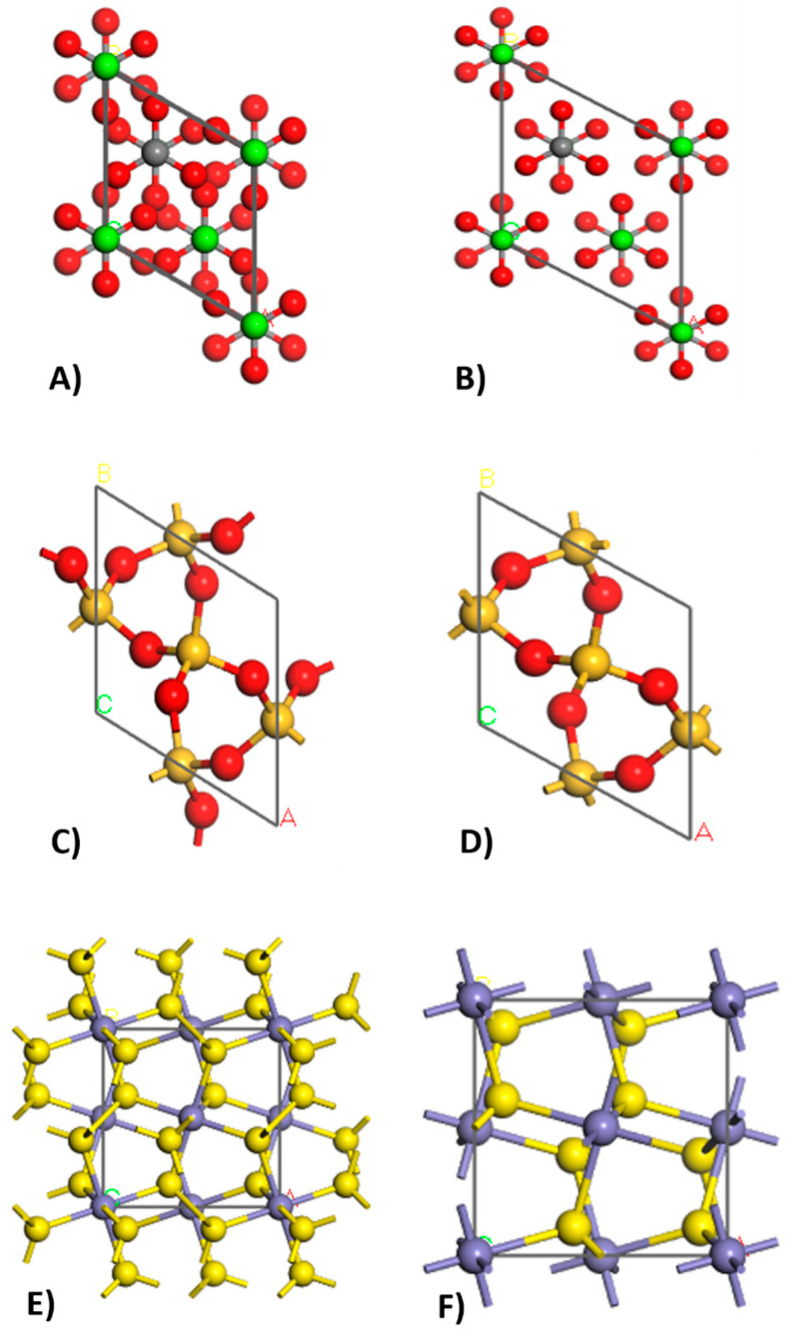
Simulated structures before geometric optimization with an initial enthalpy of 2780.34 kcal/mol to calcite (**A**), 19.79 kcal/mol to quartz (**C**), and 475.62 kcal/mol to pyrite (**E**); and simulated structures after geometric optimization with a final enthalpy of 81.39 kcal/mol to calcite (**B**), −28.03 kcal/mol to quartz (**D**), and 368.69 kcal/mol to pyrite (**F**).

**Figure 4:**
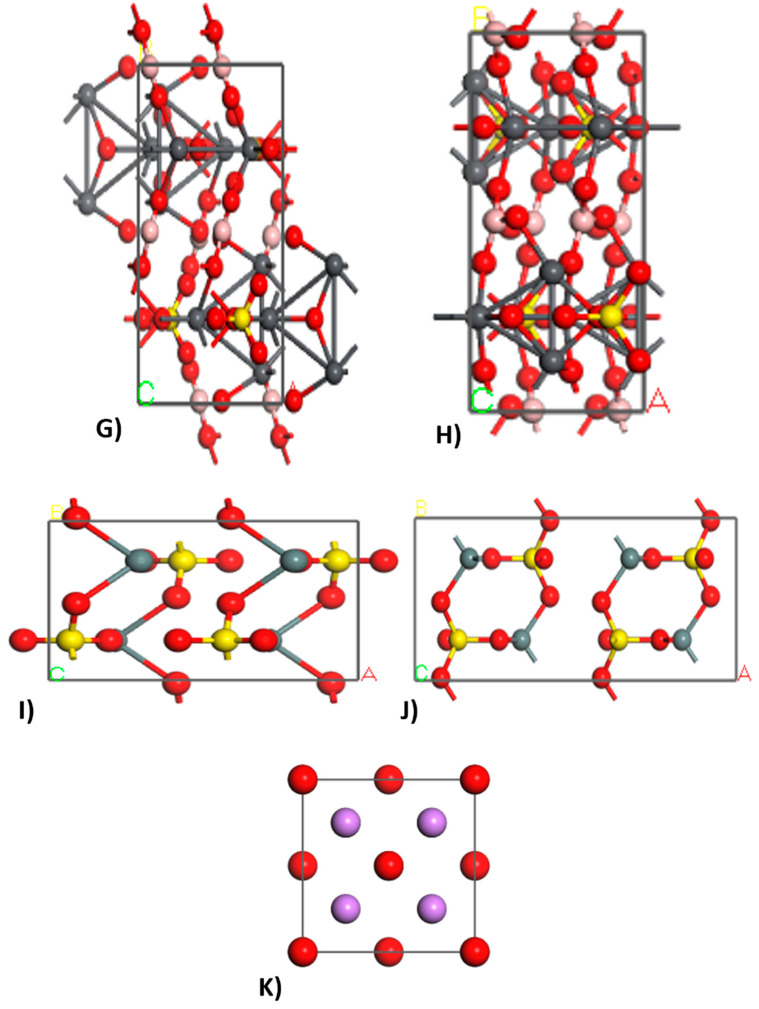
Simulated structures before geometric optimization with an initial enthalpy of 11867.75 kcal/mol to Pb_6_O_2_(BO_3_)_2_SO_4_ (**G**), 11867.75 kcal/mol to tin sulfate (**I**), and 81.78 kcal/mol to lithium oxide (**K**); and simulated structures after geometric optimization with a final enthalpy of 5466.76 kcal/mol to Pb_6_O_2_(BO_3_)_2_SO_4_ (**H**), and 5466.76 kcal/mol to tin sulfate (**J**).

Geometry optimization was carried out using the Forcite module, employing the Smart optimization algorithm and the Universal force field. This process enabled us to achieve the structures in their minimum energy state. Figure 3 and Figure 4 illustrate the structures before and after geometry optimization, highlighting differences in atomic size and arrangement.

The Universal force field (UFF) has been widely utilized in molecular dynamics (MD) simulations across various studies involving inorganic compounds, demonstrating its validity and reliability in computational modeling. The UFF is particularly advantageous due to its ability to provide a consistent framework for simulating a diverse range of materials, including metal–organic frameworks (MOFs) and other inorganic structures.

For instance, [29] conducted MD simulations using the UFF within the Forcite module of Materials Studio to investigate the growth limits of ZIF-8 thin films. Their work involved constructing defect-free models based on X-ray crystal structures, showcasing the UFF’s capability to accurately represent the structural properties of complex inorganic materials [29]. Similarly, [30] employed the UFF to calculate vibrational frequencies and temperatures of rhodochrosite crystals, further validating the method’s effectiveness in predicting the physical properties of inorganic compounds.

Moreover, the UFF has been successfully applied in studies focusing on gas separation processes. The authors of [31] utilized the UFF in their MD simulations to explore the efficiency of helium separation using a two-dimensional metal–organic framework, demonstrating the force field’s applicability in practical scenarios involving gas interactions. Additionally, [32] highlighted the importance of selecting appropriate force fields for MOFs, comparing the UFF with other generic force fields and finding it to yield reliable predictions for CO_2_ separation processes.

The versatility of the UFF is further illustrated by its application in various other studies. For example, Aly et al. optimized the molecular structures of metal complexes using the UFF, confirming its utility in quantum chemical studies [33]. In another study, [34] employed the UFF to simulate the behavior of polyether/polyamide-Ag membranes for pollutant capture, reinforcing the method’s robustness in modeling interactions in complex systems.

The UFF has demonstrated significant reliability in predicting inorganic crystal structures, particularly when benchmarked against specialized force fields. For instance, Addicoat et al. showed that Universal force field for metal–organic frameworks (UFF4MOF) parameters yield lattice parameters for MOFs comparable to those derived from dedicated force fields, indicating the UFF’s robustness in this domain [35]. Furthermore, Jaillet et al. emphasized the UFF’s versatility, as it encompasses parameterizations for all elements up to atomic number 103, making it applicable across a wide range of inorganic systems [36]. This adaptability is crucial for modeling complex interactions in materials science, as evidenced by its successful application in various studies, including those on metal complexes [36]. Additionally, the accuracy of UFF computations has been validated through comparisons with experimental data, reinforcing its utility in computational modeling of crystal structures [37]. Overall, the UFF’s comprehensive parameterization and proven performance in diverse applications underscore its value in predicting inorganic crystal structures reliably. In summary, the UFF has been validated through multiple studies that demonstrate its effectiveness in simulating the molecular dynamics of inorganic compounds and materials. Its consistent performance across diverse applications underscores its relevance in computational materials science.

### 3.4. Structural Validation 

Table 3 presents the structural validation for the optimized figures by density comparison, where coincidence can be observed for five of the six simulated structures, except for calcite. 

Errors in calculated densities, both experimental and simulation-based, can arise from several factors. For experimental densities obtained through X-ray diffraction, inaccuracies may stem from instrumental calibration, sample purity, and environmental conditions during measurement, which can affect the precision of density values [38]. Additionally, the presence of defects or imperfections in the crystal structure can lead to discrepancies between measured and theoretical densities.

In simulation-based density calculations, such as those performed using Materials Studio, errors can occur due to the choice of computational parameters, including the level of theory, basis set, and optimization algorithms used [39]. Furthermore, approximations inherent in the models, such as the generalized gradient approximation (GGA) in density functional theory, can lead to systematic errors in predicted densities [40]. Assumptions made regarding intermolecular interactions and simplifications in the simulation models can also contribute to inaccuracies [41]. Overall, a comprehensive understanding of both experimental and computational limitations is essential for interpreting density results accurately.

### 3.5. Interaction Parameter 

The interaction parameter (*χ*) was evaluated, and was taken as the basis on which the miscibility of each of the simulated crystals would be evaluated, with lithium oxide as a screen, thus obtaining the miscibility parameters presented in Table 4.

The Flory–Huggins interaction parameter *χ* can be related to the Hildebrand solubility parameter δ and expressed as a function of temperature. A small value of *χ* is generally an indicator of miscibility in a binary system [42].

## 4. Discussion

Geometric optimization, which involves reducing cell sizes and adjusting atomic arrangements, led to a significant decrease in the system’s specific enthalpy. For calcite, this change was from an initial enthalpy of 2780.34 kcal/mol (A) to a final enthalpy of 81.39 kcal/mol (B). Geometric optimization was conducted for each simulated crystal, as shown in Figure 3 and Figure 4. However, the simulated crystal for lithium oxide (K) was not geometrically optimized because the data from the database already represent the ideal crystal structure for that compound in its minimum energy state [43,44,45].

The differences observed in structural validation for the optimized figures, as measured by density comparisons, may stem from the optimization process, which includes two main stages: energy evaluation and conformation adjustment. In the energy evaluation stage, conformations and energy terms are assessed and defined. The conformation adjustment stage aims to reduce energy expression, which may be achieved through adjustment or require additional iterations. The efficiency of the optimization is determined by both the time taken to evaluate the energy expression and the number of iterations needed to reach the minimum energy state [46]. Although two full simulations were performed for each structure, no significant change in structural density was observed. To achieve the minimum energy state, it may be necessary to increase the number of iterations or vary the force field.

The interaction parameter *χ* ≥ 0 indicates a tendency for components to remain separate without mixing, as seen with the lowest values in interactions between pyrite and lithium oxide (0.6052), calcite (4.2978), and quartz (8.9556). Conversely, an *χ* ≫ 0 value suggests phase separation in the mixture, as observed for the Pb_6_O_2_(BO_3_)_2_SO_4_ compound.

In molecular dynamics simulations conducted using Materials Studio, the values of binding energies and coordination numbers (Z) are derived through a combination of computational techniques and modules available within the software. The binding energy, which quantifies the stability of a molecular system, is typically calculated using density functional theory (DFT) or force field methods. For instance, the DMol3 module in Materials Studio is frequently employed to perform DFT calculations, allowing researchers to obtain total energies and electronic properties essential for determining binding energies [47,48].

Coordination numbers, which indicate the number of nearest neighbors surrounding a central atom, are often derived from the analysis of radial distribution functions (RDFs) obtained during MD simulations. The Forcite module in Materials Studio is particularly useful for such calculations, as it allows for the simulation of molecular interactions and the extraction of structural information from the resulting trajectories [49]. By analyzing the RDF, one can determine the peak positions corresponding to the distances of nearest neighbors, which directly informs the coordination number [50]. This approach has been validated in various studies, where MD simulations have successfully modeled the solvation shells and coordination environments of ions in different solvents [50].

Furthermore, the integration of these computational techniques with advanced visualization tools in Materials Studio enhances our understanding of material properties at the atomic level. For example, the CASTEP module can be utilized for geometry optimization and electronic structure calculations, which are crucial for accurately determining both binding energies and coordination numbers in complex materials [51,52]. The combination of these methodologies allows researchers to comprehensively analyze interactions within materials, leading to insights into their mechanical and thermal properties [53].

In summary, the determination of binding energies and coordination numbers in molecular dynamics simulations using Materials Studio involves a multifaceted approach that leverages DFT calculations, force field methods, and detailed structural analysis of simulation data. This integrated methodology provides a robust framework for exploring the intricate behaviors of materials at the molecular level.

## 5. Conclusions

In conclusion, the application of molecular dynamics (MD) simulations in this study is strongly supported by key quantitative characteristics that enhance our understanding of mineral extraction processes, particularly for lithium. The identification of five mineral phases—quartz, calcite, pyrite, tin oxide, and Pb_6_O_2_(BO_3_)_2_SO_4_—highlights the complexity of the polylithionite mineral concentrate, necessitating detailed MD simulations to model their interactions. Structural optimization through precise crystallographic data, including lattice parameters and atomic coordinates, enables reliable predictions of phase behavior. Additionally, the validation of optimized structures via density comparisons provides a critical benchmark for assessing thermodynamic properties. The calculated miscibility parameters (*χ*) further illustrate significant affinities between lithium oxide and pyrite, as well as between calcite and quartz, offering valuable insights for optimizing extraction pathways. These findings collectively underscore the importance of MD simulations in advancing sustainable lithium extraction methodologies in the context of the electromobility era.

## Figures and Tables

**Table 1:** Crystallographic information obtained from XRD analysis of lithium-bearing mineral concentrate.

Phase	Formula	Space Group	Latency Parameters					
			a (Å)	b (Å)	c (Å)	α (°)	β (°)	γ (°)
Calcite	CaCO_3_	167: R-3c	4.9937	4.9937	17.0792	90	90	120
Quartz	SiO_2_	152: P3121	4.9198	4.9198	5.4018	90	90	120
Pyrite	FeS_2_	205: Pa-3	5.4279	5.4279	5.4279	90	90	90
Pb_6_O_2_(BO_3_)_2_SO_4_	B_2_O_12_Pb_6_S	62: Pnma	6.3849	11.3296	17.7568	90	90	90
Tin oxide	SnSO_4_	62: Pnma	8.6952	5.3834	6.9650	90	90	90
Lithium Oxide *	LiO_2_	Fm3̅m	4.65	4.65	4.65	90	90	90

* Information obtained from the database The Materials Project (2020) [27].

**Table 2:** Structural parameters corresponding to the atomic coordinates for each of the phases obtained in the XRD analysis.

Phase	Element	X	Y	X
Calcite (CaCO_3_)	Ca	0.000	0.000	0.000
	C	0.000	0.000	0.250
	O	0.257	0.000	0.250
Quartz (SiO_2_)	Si	0.465	0.000	0.333
	O	0.417	0.278	0.222
Pyrite (FeS_2_)	Fe	0.000	0.000	0.333
	S	0.625	0.625	0.625
Pb_6_O_2_(BO_3_)_2_SO_4_	O	0.030	0.025	0.447
	S	0.212	0.250	0.493
	O	0.167	0.650	0.974
	O	0.087	0.250	0.161
	Pb	0.224	0.250	0.292
	Pb	0.171	0.093	0.105
	O	0.164	0.250	0.571
	Pb	0.402	0.250	0.699
	B	0.079	0.508	0.748
	O	0.141	0.597	0.718
	Pb	0.645	0.590	0.102
	O	0.750	0.491	0.829
	O	0.281	0.750	0.355
	O	0.024	0.402	0.713
Tin sulfate (O_4_SSn)	Sn	0.208	0.250	0.222
	S	0.069	0.250	0.694
	O	-0.083	0.250	0.597
	O	0.194	0.250	0.550
	O	0.088	0.020	0.819
Lithium Oxide (Li_2_O)	O	0.000	0.000	0.000
	Li	0.250	0.750	0.250

**Table 3:** Structural validation of the optimized figures by density comparison.

Name	Formula	Density [g/cm^3^]	
		Experimental	Simulated
Calcite	CaCO_3_	2.703	1.697
Quartz	SiO_2_	2.643	2.708
Pyrite	FeS_2_	4.983	4.594
Pb_6_O_2_(BO_3_)_2_SO_4_	B_2_O_12_Pb_6_S	7.699	6.330
Tin sulfate	SnSO_4_	4.375	3.540
Lithium oxide	LiO_2_	1.960	1.959

**Table 4:** Interaction parameter χ  (Chi) for Li_2_O for each simulated crystal.

Base	Display	χ
Pb_6_O_2_(BO_3_)_2_SO_4_	LiO_2_	67.4689
Tin sulfate	LiO_2_	11.1287
Quartz	LiO_2_	8.9556
Calcite	LiO_2_	4.2978
Pyrite	LiO_2_	0.6052

## Data Availability

The original contributions presented in this study are included in this article. Further inquiries can be directed to the corresponding author.
